# Hyperglycemia combined *Helicobacter pylori* infection increases risk of synchronous colorectal adenoma and carotid artery plaque

**DOI:** 10.18632/oncotarget.22094

**Published:** 2017-10-26

**Authors:** Kuang-Chun Hu, Ming-Shiang Wu, Cheng-Hsin Chu, Horng-Yuan Wang, Shee-Chan Lin, Helen L. Po, Ming-Jong Bair, Chuan-Chuan Liu, Tung-Hung Su, Chi-Ling Chen, Chun-Jen Liu, Shou-Chuan Shih

**Affiliations:** ^1^ Division of Gastroenterology, Department of Internal Medicine, MacKay Memorial Hospital, Taipei, Taiwan; ^2^ Healthy Evaluation Center, MacKay Memorial Hospital, Taipei, Taiwan; ^3^ MacKay Medicine, Nursing and Management College, Taipei, Taiwan; ^4^ Graduate Institute of Clinical Medicine, National Taiwan University College of Medicine, Taipei, Taiwan; ^5^ Department of Internal Medicine, National Taiwan University Hospital, National Taiwan University College of Medicine, Taipei, Taiwan; ^6^ Department of Neurology, MacKay Memorial Hospital, Taipei, Taiwan; ^7^ Gastroenterology Division, Department of Internal Medicine, MacKay Memorial Hospital, Taitung Branch, Taiwan; ^8^ MacKay Medical College, Taipei, Taiwan

**Keywords:** synchronous, colorectal adenoma, carotid artery plaque, hyperglycemia, Helicobacter pylori

## Abstract

**Background:**

Cardiovascular disease and colorectal cancer have severe consequences to human health and may occur simultaneously or sequentially. Carotid artery plaque is a predictor of cardiovascular disease, and colorectal adenoma is a premalignant lesion of colorectal cancer. We investigated the core risk factors of carotid artery plaque and colorectal adenoma.

**Results:**

In total, 2361 subjects were enrolled. In multivariate analysis, age ≥ 60 years, male sex, BMI > 27, LDL > 130 mg/dL, HbA_1c_ ≥ 6.5%, hs-CRP > 0.3 mg/L and *H. pylori* infection were independent risk factors for synchronous colorectal adenoma and carotid artery plaque formation. In the *H. pylori*-positive and -negative groups, the proportions and odds ratio (OR) for synchronous colon adenoma and carotid artery plaque increased with increasing HbA1c. OR for synchronous colon adenoma and carotid artery plaque was significantly higher in the participants with HbA_1c_ levels of 5.7%–6.4% and HbA1c ≥ 6.5% than in those with normal HbA_1c_ in the *H. pylori*-negative group. The OR was more significant increased for *H. pylori*-positive patients when HbA_1c_ level ≥ 6.5% was 15.87 (95% CI 8.661–29.082, *p* < 0.0001).

**Materials and Methods:**

The records of 4669 subjects aged > 40 years who underwent bidirectional gastrointestinal endoscopy and carotid artery ultrasound examination on the same day or within 12 months of endoscopy examination from January 2006 to December 2015 were reviewed. All subjects had a gastric biopsy specimen tested for *Helicobacter pylori*.

**Conclusions:**

Hyperglycemia combined with H. pylori infection was an increased risk factor for synchronous colorectal adenoma and carotid artery plaque formation. Diabetes control and *H. pylori* eradication may be warranted in higher prevalence areas.

## INTRODUCTION

Over the last several decades, patients who had coronary artery disease have been found to be at increased risk of developing colon adenoma [1, 2]. The lifetime risk of acute myocardial infarction is 30% and of stroke is 40% in Westernized countries [3, 4]. These two diseases are related to arthrosclerosis and are types of cardiovascular disease (CVD). Several studies have shown that carotid artery plaque formation increased the risk of CVD [5, 6]. Colon-rectal adenoma is a premalignant lesion in colorectal cancer and develops into colorectal carcinoma (CRC) through the adenoma-to-carcinoma sequence. CRC is the third most common malignancy and the fourth most common cause of cancer-related death worldwide [7]. In a recent study, the mortality rate of CVD had decreased slightly. From 2000 to 2010, age-adjusted mortality decreased by 30% for heart disease and by 36% for stroke in the United States [8]. These findings may have been related to more public bans on smoking and lower target levels of low-density lipoprotein cholesterol and blood pressure that contributed to improved control of risk factors over time [9]. The CRC incidence and mortality rates vary widely worldwide, and stabilizing or decreasing trends is seen in highly developed countries. In developing countries, however, the CRC incidence and mortality have also increased [10]. CVD and CRC share similar risks factors, including male sex, aging, hyperglycemia, smoking, hyperlipidemia, and obesity [11, 12].

*Helicobacter pylori* infection has been associated with colon adenoma formation [13] and with an increased incidence of ischemic stroke [14, 15]. Hyperglycemic status has been associated with a higher prevalence of colon adenoma and CVD [16, 17]. This means that *H. pylori* infection and hyperglycemia may be common risk factors of CVD and CRC. Because the mortality for these two severe diseases has decreased, we can expect that patients who have survived one of these diseases may eventually develop the other. However, some patients may simultaneously develop colorectal adenoma and CVD. For physicians, the ability to “see the future” is valuable [18]. It is challenging to predict if a patient will develop CRC after developing CVD, especially in the early stage, such as colon adenoma and carotid artery plaque. Previous studies have shown a close relationship between CVD and CRC [1, 2] and that these two diseases shared similar risk factors such as gender, aging, smoking, hyperglycemia, and *H. pylori* infection [11–15]. In these factors, hyperglycemia and *H. pylori* infection have more possibility to be modified by medical intervention and can be treated with appropriate medicine. In our previous study, we found that *H. pylori* infection and hyperglycemia were involved in colon adenoma formation and had a synergistic effect [19]. This result hinted us that these two risk factors combined may affect other serious diseases such as CVD. There are few reports on more detailed interactions between the risk factors of these diseases. There have been no reports on the types of patients at higher risk for development of these two severe diseases either simultaneously or sequentially. Here we investigated the core risk factors of synchronous colon adenoma and carotid artery plaque.

## RESULTS

### Demographics of colon adenoma and carotid artery plaque statuses

According to the colonoscopy and carotid artery ultrasound examination results, we separated the participants into three groups: (a) colon adenoma combined with carotid artery plaque formation, (b) either colon adenoma or carotid artery plaque, (c) no colon adenoma and no carotid artery plaque. There were significant differences in all variables except in total cholesterol among the three groups (Table 1). The data also demonstrated trends toward increased age, male sex, blood pressure, glucose AC level, HbA_1c_, triglycerides, white blood cell count, high-sensitivity C-reactive protein (hs-CRP) > 0.3 mg/L, *H. pylori* infection, smoking, use of anti-platelet agents, and use of anti-lipid agents in the participants with colon adenoma combined with carotid artery plaque formation. The demographics for the only colon adenoma or only carotid artery plaque outcomes are presented in Supplementary Table 1A and 1B.

**Table 1:** Demographics of colon adenoma and carotid artery plaque status

Variable	Synchronous adenoma and plaque (*N* = 224)	Either adenoma or plaque(*N* = 812)	No adenoma and No plaque (*N* = 1325)	*p*-value
**Age, mean (SD), year**	59.24 (8.52)	56.20 (9.83)	50.33 (9.75)	< .0001
**Sex (male %)**	178 (79.46)	579 (71.31)	796 (60.12)	< .0001
**BMI, mean (SD), kg/m**^2^	25.46 (3.70)	25.18 (7.25)	24.19 (3.41)	< .0001
**HbA1c, mean (SD), %**	6.20 (1.23)	5.90 (0.97)	5.71 (0.75)	< .0001
**Systolic blood pressure, mean (SD) mm Hg**	136.86 (134.89)	126.17 (46.90)	120.08 (37.58)	< .0001
**Diastolic blood pressure, mean (SD) mm Hg**	81.75 (50.60)	78.35 (23.84)	75.56 (19.62)	0 .0009
**Glucose AC, mean (SD), mg/dL**	111.39 (32.97)	103.32 (25.07)	98.97(20.60)	< .0001
**Total cholesterol, mean (SD), mg/dL**	206.26 (37.77)	204.96 (38.05)	202.80 (36.14)	0 .2533
**Triglyceride, mean (SD), mg/dL**	153.25 (77.64)	150.37 (95.38)	134.94 (80.96)	< .0001
**LDL, mean (SD), mg/dL**	141.02 (36.04)	137.80 (35.75)	134.66 (33.96)	0.0149
**WBC count, mean(SD), x 10**^3^**/μL**	6.45 (1.74)	6.48 (1.89)	6.17 (1.76)	0.0004
**Plasma hs-CRP > 0.3 mg/L, no. (%)**	38(29.69)	104 (22.41)	119 (16.08)	0.0003
**Smoking, no. (%)**	61 (27.23)	211 (25.99)	281 (21.22)	0.0154
**Anti-platelet agent used, no. (%)**	18 (8.04)	46 (5.67)	51 (3.85)	0.0118
**Anti-lipid agent used, no. (%)**	39 (17.41)	118 (14.53)	118 (8.92)	< .0001
**DM control agent used, no. (%)**	44 (19.64)	77 (9.52)	97 (7.34)	< .0001
**Hypertension control agent used, no. (%)**	90 (40.18)	246 (30.41)	216 (16.35)	< .0001
**Hb A**_1c_ **level ≥ 6.5%, no. (%)**	54 (24.11)	125 (15.39)	97 (7.32)	< .0001
***H. pylori*-positive, no. (%)**	112 (50)	366 (45.07)	414 (31.25)	< .0001

BMI: body mass index; HbA1c, glycated hemoglobin; Glucose AC: fasting blood glucose; LDL: Low-density lipoprotein; hs-CRP, high-sensitivity C-reactive protein DM: diabetes mellitus no.: number.

### Univariate analysis and multivariate logistic regression for predictors of colorectal adenoma combined with carotid artery plaque

Table 2 shows the factors that were subjected to univariate and multivariate analyses. Multivariate logistic regression analysis showed that age ≥ 60 years, male sex, BMI > 27, LDL > 130 mg/dL, HbA_1c_ ≥ 6.5%, hs-CRP > 0.3 mg/L, *H. pylori* infection, and hypertension control agents used were independent risk factors for synchronous colorectal adenoma and carotid artery plaque formation. Of these factors, LDL, HbA_1c_ level, and *H. pylori* infection status could be adjusted. Age and sex obviously could not be changed. The univariate and multivariate analyses for the potential risk factors of only colon adenoma or only carotid artery plaque formation are shown in Supplementary Table 2A and 2B.

**Table 2:** Univariate analysis and multivariate logistic regression for predictors of colon adenoma combined with carotid artery plaque

	Synchronous adenoma and plaque	No adenoma and no plaque	Univariate analysis		Multivariate logistic regression	
			OR (95% CI)	*p*-value	Adjusted OR (95% CI)	*p*-value
**Age ≥ 60 years**	99	208	4.25 (3.141, 5.750)	< .0001	2.938 (1.865, 4.631)	< .0001
**Male, sex**	178	796	2.567 (1.823, 3.614)	< .0001	3.013 (1.787, 5.08)	< .0001
**BMI > 27**	67	234	1.988 (1.445, 2.735)	< .0001	1.227 (0.752, 2.001)	0.4126
**Systolic_pressure ≥140 mm Hg**	50	148	2.285 (1.598, 3.269)	< .0001	1.321 (0.770, 2.269)	0.3123
**Smoking**	61	281	1.389 (1.006, 1.918)	0.0456	1.246 (0.748, 2.076)	0.3980
**Anti-platelet agent used**	18	51	2.179 (1.248, 3.804)	0.0061	0.904 (0.404, 2.022)	0.8068
**Anti-lipid agent used**	39	118	2.153 (1.452, 3.191)	0.0001	1.761 (0.948, 3.270)	0.0731
**Hypertension control agent used**	90	216	3.436 (2.535, 4.658)	< .0001	1.917 (1.192, 3.084)	0.0073
**DM control agent used**	44	97	3.087 (2.092, 4.556)	< .0001	1.279 (0.607, 2.695)	0.5179
**LDL > 130 mg/ dL**	134	694	1.406 (1.049, 1.884)	0.0224	1.576 (1.014, 2.448)	0.0432
**WBC count, × 10**^3^**/μL**	221	1298	1.089 (1.008, 1.176)	0.0305	0.925 (0.816, 1.049)	0.2228
**Plasma hs-CRP** **> 0.3 mg/L**	38	119	2.203 (1.438, 3.377)	0.0003	1.848 (1.118, 3.056)	0.0166
**Hb A**_1c_ **≥ 6.5%**	54	97	4.021 (2.779, 5.819)	< .0001	2.263 (1.134, 4.517)	0.0205
***H. pylori*-positive**	112	414	2.200 (1.652, 2931)	< .0001	2.193 (1.432, 3.358)	0.0003

BMI: body mass index; HbA1c, glycated hemoglobin; LDL: Low-density lipoprotein; hs-CRP, high-sensitivity C-reactive protein DM: diabetes mellitus.

### Relationships between HbA_1c_ level and H. pylori infection status and colon adenoma and carotid artery plaque

According to the American Diabetes Association [20], the HbA_1c_ level may have different clinical meanings. We investigated the relationship between *H. pylori* infection status combined with HbA1c level and colon adenoma and carotid artery plaque. In the *H. pylori-*positive participants, the percentage of participants with no adenoma and no plaque decreased as the HbA_1c_ level increased; conversely, this proportion increased in the participants with adenoma combined with plaque as the HbA_1c_ level increased. This trend was also observed in the *H. pylori-*negative participants (Table 3).

**Table 3:** Relationships among colon adenoma combined with carotid artery plaque and different HbA_1c_ levels and H. *pylori* infection statuses

	*H. pylori*-negative (*N* = 1469)					*H. pylori*-positive (*N* = 892)			
HbA_1c_ level	≤ 5.6	5.7∼6.4	≥ 6.5	*p*-value	≤ 5.6		5.7∼6.4	≥ 6.5	*p-*value
No adenoma and No plaque	517 (65.69%)	322 (60.87%)	72 (47.06%)	< .0001	234 (55.19%)		155 (44.93%)	25 (20.33%)	< .0001
Only adenoma/ No plaque	131 (16.65%)	95 (17.96%)	29 (18.95%)		97 (22.88%)		68 (19.71%)	39 (31.71%)	
No adenoma/ Only plaque	96 (12.20%)	64 (12.10 %)	31 (20.26%)		61 (14.39)		75 (21.74%)	26 (21.14%)	
Synchronous adenoma and plaque	43 (5.46%)	48 (9.07%)	21 (13.70%)		32 (7.55%)		47 (13.62%)	33 (26.83%)	
Total	787 (100%)	529 (100%)	153 (100%)		424 (100%)		345 (100%)	123 (100%)	

### Odds ratio of synchronous colorectal adenoma and carotid artery plaque for different HbA_1c_ levels and H. pylori infection statuses

**Figure 1:**
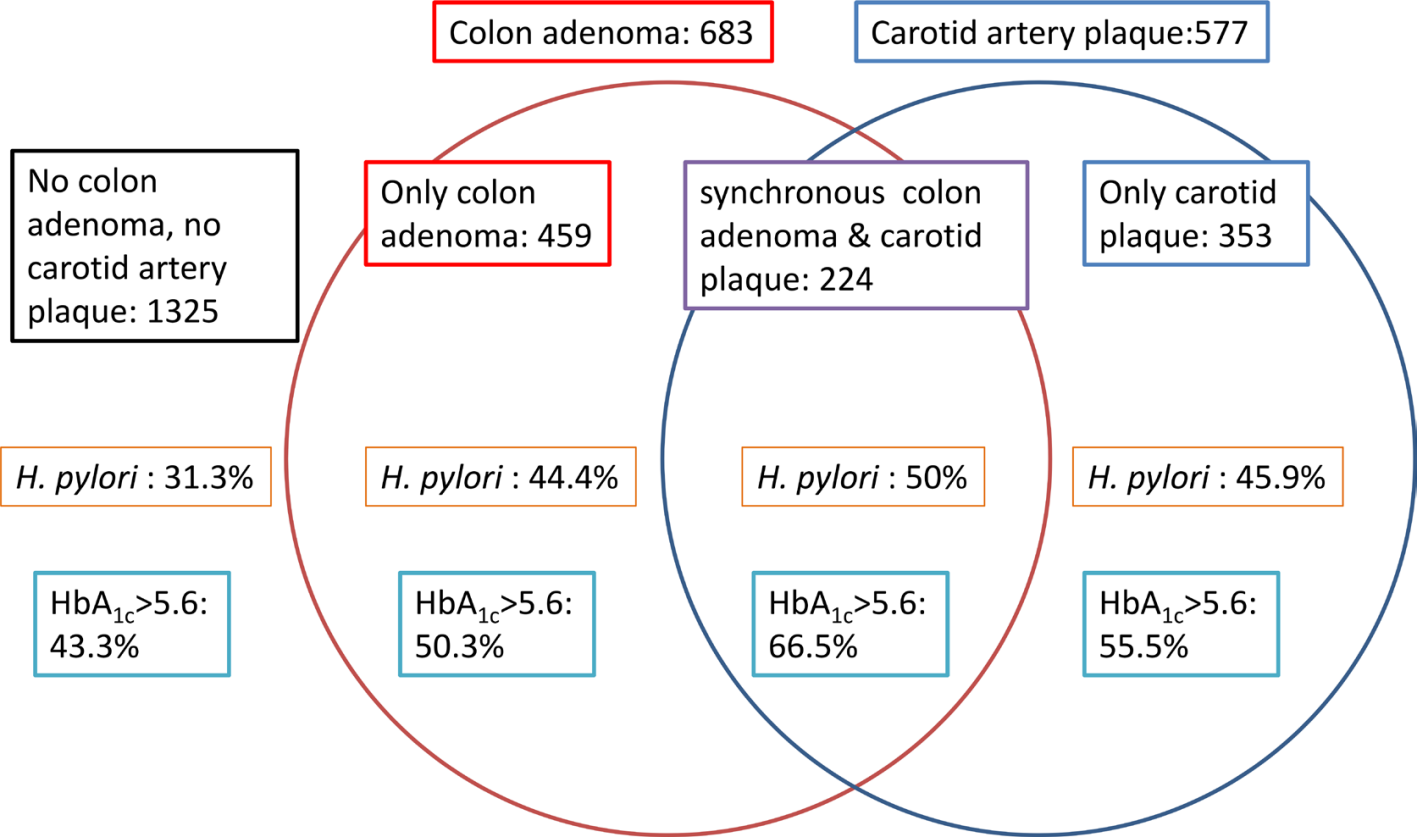
Distributions of colon adenoma combined with carotid artery plaque in *H. pylori* infection status and HbA_1c_ abnormal percent

In the *H. pylori-*negative group, OR for synchronous colon adenoma and carotid artery plaque was significantly higher in the participants with HbA_1c_ levels of 5.7%–6.4% (OR, 1.792; 95% CI, 1.161–2.767; *p* = 0.0085) and ≥ 6.5% (OR, 3.507; 95% CI, 1.969–6.245; *p* < 0.0001) than in the participants with normal HbA_1c_. OR for the *H. pylori-*positive participants with HbA_1c_ levels of 5.7%–6.4% was even higher (OR, 3.646; 95% CI, 2.323–5.732; *p* < 0.0001). Compared with the participants with normal HbA_1c_ who were *H. pylori-*negative, those who had both HbA_1c_ ≥ 6.5% and *H. pylori* infection had an OR of 15.870 (95% CI, 8.661–29.082; *p* < 0.0001) (Table 4). Similar results are presented in Figure 1. When the participants suffered from colon adenoma and carotid plaque, the proportions of participants with HbA_1c_ > 5.6% and *H. pylori* infection were higher than those in the participants with either colon adenoma or carotid plaque or no colon adenoma or carotid artery plaque.

**Table 4:** Odds ratios of colon adenoma combined with carotid plaque formation for different HbA_1c_ levels and *H. pylori* infection statuses

		Synchronous adenoma and plaque	No adenoma and no plaque	Adenoma combine plaque OR	
HbA_1c_ level	*H. pylori* infection status			OR (95% CI)	*p*-value
HbA_1c_ ≤ 5.6%	*H. pylori-*negative	43	517	1	
	*H. pylori-*positive	32	234	1.644 (1.014, 2.665)	0.0436
5.7% ≤ HbA_1c_ ≤ 6.4%	*H. pylori-*negative	48	322	1.792 (1.161, 2.767)	0.0085
	*H. pylori-*positive	47	155	3.646 (2.323, 5.723)	< .0001
HbA_1c_ ≥ 6.5 %	*H. pylori-*negative	21	72	3.507 (1.969, 6.245)	< .0001
	*H. pylori-*positive	33	25	15.870 (8.661, 29.082)	< .0001

## DISCUSSION

Our previous study had demonstrated that hyperglycemia and *H. pylori* infection have a synergistic effect on colorectal adenoma risk [19]. This study found that *H. pylori* infection combined with elevated HbA_1c_ level was a risk factor for development of colon adenoma with carotid artery plaque. With public health and medical advancements, CRC and CVD mortalities have decreased in some developed areas [7, 8]. We expect that the number of patients who will suffer from these two diseases either simultaneously or sequentially will increase. Physicians should identify patients who are at a high risk of developing CVD and CRC. Colon adenoma is a premalignant lesion that develops into CRC through the adenoma-to-carcinoma sequence. Carotid artery plaque also is strongly associated with CVD. Here we investigated the connection between colon adenoma and carotid artery plaque and identified risk factors for co-development of these diseases. Our results showed that the participants who had *H. pylori* infection and HbA_1c_ > 6.5% were 16 times more likely to develop colon adenoma combined with carotid artery than those without *H. pylori* infection and HbA_1c_ < 5.6%.

Regarding the mechanism of atherosclerosis, past studies have shown that bacterial endotoxins, apoptotic cell fragments, and oxidized LDL particles are all taken up through toll-like receptors into macrophages and are followed by induction of a signal cascade that leads to cell activation [21, 22]. Progression to tissue inflammation, damage, and plaque formation on the blood vessel wall then occurs [23]. Kaplan M et al. have shown that atherosclerotic plaques of the carotid artery had *H. pylori* DNA, as detected by polymerase chain reaction [24]. Grivennikov et al. studied the role of microbiota in inflammation of the gut and found that when toll-like receptors were stimulated by bacterial products, such as endotoxin, IL-23 increased and acted on the downstream cells, including the lymphocytes. Finally, the inflammatory process is activated with subsequent increases in IL-17 and IL-6. IL-17 activates the signal transducer and activator of transcription three pathway, which promotes cell proliferation and survival, and finally induces tumorigenesis [25]. Endotoxin is a complex that contains lipopolysaccharide and elicits strong immune responses in humans. This outer membrane forms gram-negative bacteria. *H. pylori* is a gram-negative bacterium that infects approximately 50% of the world’s population [26, 27]. The above studies suggest that chronic infection status, such as *H. pylori,* may be a co-promoter that triggers formation of colon adenoma and carotid artery plaque.

The relationship between hyperglycemia status and CVD has been well studied in many reports, [28, 29] and correlation between hyperglycemia and colon adenoma also has been reported [17]. The mechanism of hyperglycemia-induced CVD is thought to be related to oxidative status, and hyperinsulinemia resulting from insulin resistance is thought to possibly promote carcinogenesis directly by stimulating colonic cell growth [30]. In hyperinsulinemia status, the insulin-like growth factor 1 (IGF-1) level increases, which enhances cell proliferation and inhibits apoptosis, thus promoting tumor growth [31]. Of our participants, 9.2% accepted DM control agents treatment due to diabetes. This situation may decrease the association between elevated HbA_1c_ levels and carotid artery plaque formation. Our results demonstrated that the higher HbA_1c_ was an independent factor of synchronous colon adenoma and carotid artery plaque formation.

A similar mechanism underlying carotid artery plaque and colon adenoma formation may be related to chronic inflammation [23, 25]. Association between hs-CRP and CVD was well discussed in previous study. Chiu et al. also found that elevated C-reactive protein levels were associated with colon adenoma [32, 33]. In our study, hs-CRP > 0.3 mg/L was also one of the risk factors for synchronous colorectal adenoma and carotid artery plaque (OR: 1.848; 95% CI: 1.118, 3.056) compared with no colon adenoma and no carotid artery plaque participants (Table 2). Hyperglycemic status and *H. pylori* infection have been associated with the chronic inflammation process [27, 34]. A possible mechanism underlying synchronous colon adenoma and carotid artery plaque is related to hyperglycemia that changes gut permeability and damages the gut barrier. Gut microbiota products, such as endotoxin, would be able to transfer more easily from the gut into the host. Endotoxin would promote atherosclerosis and colon neoplasm formation [23, 25, 35]. Hyperglycemia and hyperinsulinemia also increase IGF-1 levels, which may promote tumor growth by enhancing cell proliferation and inhibiting apoptosis [30]. When *H. pylori* infection is present with hyperglycemia, the probability of synchronous colon adenoma and carotid artery plaque should increase.

Low-density lipoprotein (LDL) is one of the major independent risk factors of CVD. The prevention of CVD by a lower LDL level has been well studied [36]. Bayerdorffer et al. first investigated the relation between the serum LDL cholesterol level and the frequency of colorectal adenomas; they found that LDL levels were positively associated with colon adenoma frequency [37]. Usage of statins was associated with a 47% relative reduction in the risk of colorectal cancer [38]. Recently, some scientists found that colorectal cancer tissue produces an excess of ox-LDL, suggesting a close correlation between lipid dysfunction and tumor formation. Their study showed lectin-like oxidized LDL receptor-1 is involved in several mechanisms closely linked with malignant transformation [39]. The possible mechanism of LDL level change induced colon adenoma and CVD may through increased oxidative stress and then involves NF-κB pathway, an oncogenic protein that regulates the transcription of several genes involved in immune and inflammatory responses as well as endothelial dysfunction. NF-κB also plays a role in atherosclerosis and finally triggers colon adenoma and carotid artery plaque formation [40].

In Table 1, the LDL level in the synchronous colon adenoma and carotid artery plaque groups was higher than in the control group. After multivariate logistic regression analysis, LDL > 130 mg/dL was also considered an independent risk factor of combined colon adenoma and carotid plaque formation. Not only systolic blood pressure but also diastolic blood pressure of synchronous adenoma and plaque participants was significant higher than other two groups. But after multivariate logistic regression analysis, OR of systolic blood pressure > 140 mmHg was no more significant. We suspected that this factor may be confounded due to hypertension control agents used. Conversely, the elevated HbA_1c_ levels in participants were significant higher in synchronous adenoma and plaque groups. Even in multivariate logistic regression analysis, HbA_1c_ > 6.5% was one of the independent risk factors of synchronous adenoma and plaque formation. Our study showed that participants with both colon adenoma and carotid artery plaque had a higher proportion of *H. pylori* infection and hyperglycemia than those without colon adenoma and carotid artery plaque. The patients with HbA_1c_ > 6.5% combined with *H. pylori* infection had a 16 times higher risk for simultaneous development of colon adenoma and carotid artery plaque than those without *H. pylori* infection and HbA_1c_ < 5.6%.

There are two reasons for physicians to highlight this issue. First, CVD and CRC mortality has decreased in some countries, so patients who survive one of these diseases may develop the other. Therefore, if we identify patients who have CVD or CRC and hyperglycemia combined with *H. pylori* infection, we may be able to predict the possibility of developing the other disease. Second, the prevalence of diabetes has increased rapidly in recent decades, especially in developing countries, and most of these areas have observed a higher prevalence of *H. pylori* infection. [41, 42] Consequently, diabetes-related complications may also increase in the future along with the prevalence of synchronous colon adenoma with carotid artery plaque.

This study has some limitations. First, this was a retrospective observational study that only included patients with relatively high incomes; they also may have been healthier and may not be representative of the general population. Second, there was an interval between the colonoscopy and carotid artery ultrasound survey for approximately 25% of the participants and hs-CRP level was obtained in only 56% of participants because they were healthy at their check-up and/or had financial restrictions, so bias may have occurred. Third, we hypothesized that endotoxin may have a major role in inducing colon adenoma and carotid artery plaque. However, *H. pylori* is not alone in the gram-negative gut microbiota. Further study is needed to determine the relationships among *H. pylori* infection, gut microbiota, and endotoxin.

## MATERIALS AND METHODS

### Patients

We retrospectively analyzed the data of 4669 subjects who had undergone routine checkups at MacKay Memorial Hospital, Taipei, Taiwan, between January 2006 and December 2015. Asymptomatic individuals who had undergone bidirectional endoscopy [complete colonoscopy, esophago-gastroduodenoscopy (EGD)] on the same day as part of a health check-up were enrolled for analysis. Carotid artery ultrasound survey was also arranged on the same day or within 12 months of colonoscopy when the participants accepted annual physical checkups. The inclusion criteria were adult patients aged > 40 years who underwent both screening colonoscopy and EGD for detection of *H. pylori* infection status by urease test. We excluded patients who (a) had proven CRC, (b) previously had proven acute myocardial infarction or stoke, (c) had a high risk of developing CRC (i.e., diagnosed cases of inflammatory bowel syndrome, positive family history of polypoid syndromes, and prior history of carcinoma), (d) had undergone incomplete colonoscopy or inadequate preparation for colonoscopy, (e) were inoperative or could not accept carotid artery ultrasound examination, (f) lacked data regarding *H. pylori* urease test examination of gastric biopsy specimens or basic blood test samples, and (g) had accepted repeated EGD and colonoscopy in annual physical examinations. After excluding 2308 subjects, a total of 2361 study participants (1553 males and 808 females) were enrolled for further study.

### Scanning protocol and definition of carotid artery lesion in ultrasound examination

Ultrasonography of the common carotid artery, carotid bifurcation, and internal carotid artery of the left and right carotid arteries was performed using a 7.5-MHz linear-array transducer (ATL Ultra-Mark IV). On a longitudinal, two-dimensional ultrasound image of the carotid artery, the anterior (near) and posterior (far) walls of the carotid artery are displayed as two bright white lines separated by a hypoechogenic space. All participants were examined by experienced ultrasonographers. The images were stored using the cine function during the electrocardiographic R-wave in the cardiac cycle. Frozen images of the arterial wall were saved, and measurements were subsequently performed on the stored digital images. Analyses were performed using the AMS program package [43]. A plaque was defined as a distinct area with an I–M thickness of > 50% thicker than neighboring sites [44].

### Scanning protocol and definition of *H. pylori* infection and colon lesion used in examinations

*H. pylori* infection was detected using the biopsy urease test (CLO test, Pronto Dry, Gastrex, Poland) using standard EGD with gastrofibroscopes (GIFQ260, Olympus Optical, Tokyo Japan). A specimen for biopsy urease testing of each subject was taken from the gastric antrum using biopsy forceps and assessed within 60 min. The agar color of the biopsy urease testing turned from yellow to red when the biopsy specimen was infected with *H. pylori*, which contained intra-cytoplasmic urease. The colonoscope (CF Q260AL; Olympus Optical, Tokyo Japan), operated by experienced gastroenterologists, was inserted from the anus up to the ileocecal area. Larger (> 0.5 cm) polyps were removed with standard polypectomy snares whereas smaller (< 0.5 cm) polyps were removed with a biopsy forceps. We classified colonoscopic findings into three subgroups: (a) polyp-free, (b) hyperplastic polyps, and (c) adenomatous polyps (tubular adenoma or tubularvillous adenoma) [45]. Analytical findings, such as juvenile or inflammatory polyps, lipomas, lymphoid aggregates, and chronic nonspecific inflammation, were regarded as normal mucosa.

### Clinical data collection and questionnaire

Clinical data included levels of fasting plasma glucose AC, hemoglobin A1C (HbA_1c_), triglyceride (TG), low-density lipoprotein (LDL), white blood cell count, and high-sensitivity C-reaction protein (hs-CRP) that were obtained from the participants on the same health check-up day as when the EGDs and colonoscopies were performed. Carotid artery ultrasound examination data were collected on the same day or within 12 months of the endoscopy examination for the participants who accepted an annual healthy examination. The participants were considered to have pre-diabetes and type 2 DM on the basis of the latest American Diabetic Association criteria [20]. To highlight the relationship between type 2 DM and colon polyps, we restricted our participants to different HbA_1c_-level groups for further analysis: (a) Normal: HbA_1c_ ≤ 5.6%, (b) Pre-diabetes: 5.7% ≤ HbA_1c_ ≤ 6.4%, and (c) Diabetes: HbA_1c_ ≥ 6.5 %. Baseline characteristics (age, gender, height, weight, blood pressure, personal medical history and current medicine use, family history of first degree-relatives, and smoking) were obtained from a questionnaire completed at the time of the examination. The study was approved by the MacKay Memorial Hospital Institutional Review Board (12MMHIS163).

### Statistical analysis

The following variables were recorded for each subject: age, sex, body mass index (BMI), HbA_1c_, lipid levels, smoking status, family history of colorectal cancer, and *H. pylori* status. The presence, size, and location of colorectal adenomas were recorded. A *t* test was applied for continuous variables when the data fit a normal Gaussian distribution. We used the *t* test to compare continuous variables for *H. pylori-*positive and -negative participants. Data for continuous variables were expressed as the mean ± SD. Categorical variables were tested using the ANOVA test and expressed as numbers (percentage). Unadjusted odds ratios (ORs) with 95% confidence intervals (CIs) were computed for potential predictors of colorectal polyps and adenomas. Multivariate logistic regression analysis was applied to compute the adjusted OR (95% CI) for predictors of colorectal polyps and adenomas. All variables with *p* < 0.2 on univariate analysis were selected for multivariate logistic regression. The final model was developed using a stepwise backward approach. All variables with *p* < 0.05 were considered to be statistically significant and remained in the final model. All analyses were performed using SAS version 9.1 (SAS Institute, Cary, NC).

## CONCLUSIONS

*H. pylori* infection combined with hyperglycemia may be a core risk factor of synchronous colorectal adenoma and carotid artery plaque formation. Given the increasing prevalence of diabetes, especially in areas with a higher prevalence of *H. pylori* infection and lower mortality for CVD and colorectal cancer, an increase in the incidence of synchronous or sequential development of these two diseases is expected. More aggressive diabetes control and *H. pylori* eradication may be warranted in these areas.

## SUPPLEMENTARY MATERIALS TABLES



## Abbreviations

HbA_1c_: glycosylated hemoglobin
hs-CRP: High-sensitivity C-reactive Protein
IGF-1: insulin-like growth factor 1
EGD: esophagogastroduodenoscopy
FPG: fasting plasma glucose
BMI: body mass index
CI: confidence intervals
CVD: cardiovascular disease
CRC: colorectal cancer
OR: odds ratio
IL: interleukin
LDL: low-density lipoprotein

## References

[1] Chan AO, Jim MH, Lam KF, Morris JS, Siu DC, Tong T, Ng FH, Wong SY, Hui WM, Chan CK, Lai KC, Cheung TK, Chan P (2007). Prevalence of colorectal neoplasm among patients with newly diagnosed coronary artery disease. JAMA.

[2] Lee JY, Hong SN, Kim JH, Choe WH, Lee S, Sung IK, Park HS, Shim CS (2013). Risk for coronary heart disease increases risk for colorectal neoplasm. Clin Gastroenterol Hepatol.

[3] Seshadri S, Wolf PA (2007). Lifetime risk of stroke and dementia: current concepts, and estimates from the Framingham Study. Lancet Neurol.

[4] Lloyd-Jones DM, Leip EP, Larson MG, D'Agostino RB, Beiser A, Wilson PW, Wolf PA, Levy D (2006). Prediction of lifetime risk for cardiovascular disease by risk factor burden at 50 years of age. Circulation.

[5] Schmidt C, Fagerberg B, Wikstrand J, Hulthe J (2003). Multiple risk factor intervention reduces cardiovascular risk in hypertensive patients with echolucent plaques in the carotid artery. Journal of Internal Medicine.

[6] Biasi GM, Froio A, Diethrich EB, Deleo G, Galimberti S, Mingazzini P, Nicolaides AN, Griffin M, Raithel D, Reid DB, Valsecchi MG (2004). Carotid plaque echolucency increases the risk of stroke in carotid stenting: The imaging in carotid angioplasty and risk of stroke study (ICAROS) study. Circulation.

[7] Tenesa A, Dunlop MG (2009). New insights into the aetiology of colorectal cancer from genome-wide association studies. Nat Rev Genet.

[8] Murphy SL, Xu J, Kochanek KD (2013). Deaths: final data for 2010. National vital statistics reports: from the Centers for Disease Control and Prevention, National Center for Health Statistics, National Vital Statistics System.

[9] Sidney S, Quesenberry CP, Jaffe MG, Sorel M, Nguyen-Huynh MN, Kushi LH, Rana JS (2016). Recent trends in cardiovascular mortality in the United States and public health goals. JAMA cardiology.

[10] Arnold M, Sierra MS, Laversanne M, Soerjomataram I, Jemal A, Bray F (2017). Global patterns and trends in colorectal cancer incidence and mortality. Gut.

[11] Winawer SJ (1999). Natural history of colorectal cancer. Am J Med.

[12] Genest Jr J, Cohn JS (1995). Clustering of cardiovascular risk factors: targeting high risk individuals. Am J Cardiol.

[13] Sonnenberg A, Genta RM (2013). Helicobacter pylori is a risk factor for colonic neoplasms. American journal of gastroenterology.

[14] Wang ZW, Li Y, Huang LY, Guan QK, Xu DW, Zhou WK, Zhang XZ (2012). Helicobacter pylori infection contributes to high risk of ischemic stroke: evidence from a meta-analysis. Journal of neurology.

[15] Huang WS, Tseng CH, Lin CL, Tsai CH, Kao CH (2014). Helicobacter pylori infection increases subsequent ischemic stroke risk: a nationwide population-based retrospective cohort study. QJM.

[16] Stratton IM, Adler AI, Neil HA, Matthews DR, Manley SE, Cull CA, Hadden D, Turner RC, Holman RR (2000). Association of glycaemia with macrovascular and microvascular complications of type 2 diabetes (UKPDS 35): prospective observational study. BMJ.

[17] Tseng PH, Lee YC, Chiu HM, Chen CC, Liao WC, Tu CH, Yang WS, Wu MS (2012). Association of diabetes and HbA1c levels with gastrointestinal manifestations. Diabetes care.

[18] Robertson DJ (2013). Prediction models for advanced neoplasia: risky business. Clin Gastroenterol Hepatol.

[19] Hu KC, Wu MS, Chu CH, Wang HY, Lin SC, Liu SC, Liu CC, Su TH, Chen CL, Liu CJ, Shih SC (2017). Synergistic effect of hyperglycemia and Helicobacter pylori infection status on colorectal adenoma risk. J Clin Endocrinol Metab.

[20] American Diabetes Association (2010). Diagnosis and classification of diabetes mellitus. Diabetes Care.

[21] Janeway CA, Medzhitov R (2002). Innate immune recognition. Annu Rev Immunol.

[22] Edfeldt K, Swedenborg J, Hansson GK, Yan ZQ (2002). Expression of toll-like receptors in human atherosclerotic lesions: a possible pathway for plaque activation. Circulation.

[23] Hansson GK (2005). Inflammation, atherosclerosis, and coronary artery disease. N Engl J Med.

[24] Kaplan M, Yavuz SS, Cinar B, Koksal V, Kut MS, Yapici F, Demirtas MM (2006). Detection of Chlamydia pneumoniae and Helicobacter pylori in atherosclerotic plaques of carotid artery by polymerase chain reaction. International journal of infectious diseases.

[25] Grivennikov SI, Wang K, Mucida D, Stewart CA, Schnabl B, Jauch D, Taniguchi K, Yu GY, Österreicher CH, Hung KE, Datz C, Feng Y, Fearon ER (2012). Adenoma-linked barrier defects and microbial products drive IL-23/IL-17-mediated tumour growth. Nature.

[26] Matysiak-Budnik T, Megraud F (1997). Epidemiology of Helicobacter pylori infection with special reference to professional risk. J Physiol Pharmacol.

[27] Kusters JG, van Vliet AHM, Kuipers EJ (2006). Pathogenesis of Helicobacter pylori Infection. Clinical Microbiology Reviews.

[28] Laakso M (1999). Hyperglycemia and cardiovascular disease in type 2 diabetes. Diabetes.

[29] Selvin E, Marinopoulos S, Berkenblit G, Rami T, Brancati FL, Powe NR, Golden SH (2004). Meta-analysis: glycosylated hemoglobin and cardiovascular disease in diabetes mellitus. Annals of internal medicine.

[30] Giovannucci E (2001). Insulin, insulin-like growth factors and colon cancer: a review of the evidence. J Nutr.

[31] Ma J, Pollak MN, Giovannucci E, Chan JM, Tao Y, Hennekens CH, Stampfer MJ (1999). Prospective study of colorectal cancer risk in men and plasma levels of insulin-like growth factor [IGF]-I and IGF-binding protein-3. J Natl Cancer Inst.

[32] Pearson TA, Mensah GA, Alexander RW, Anderson JL, Cannon RO, Criqui M, Fadl YY, Fortmann SP, Hong Y, Myers GL, Rifai N, Smith SC, Taubert K (2003). Markers of inflammation and cardiovascular disease. Circulation.

[33] Chiu HM, Lin JT, Chen TH, Lee YC, Chiu YH, Liang JT, Shun CT, Wu MS (2008). Elevation of C-reactive protein level is associated with synchronous and advanced colorectal neoplasm in men. Am J Gastroenterol.

[34] Brownlee M (2005). The pathobiology of diabetic complications. Diabetes.

[35] Kang M, Edmundson P, Araujo-Perez F, McCoy AN, Galanko J, Keku TO (2013). Association of plasma endotoxin, inflammatory cytokines and risk of colorectal adenomas. BMC cancer.

[36] Grundy SM, Balady GJ, Criqui MH, Fletcher G, Greenland P, Hiratzka LF, Houston-Miller N, Kris-Etherton P, Krumholz HM, LaRosa J, Ockene IS, Pearson TA, Reed J (1998). Primary prevention of coronary heart disease: guidance from Framingham: a statement for healthcare professionals from the American Heart Association’s Task Force on Risk Reduction. Circulation.

[37] Bayerdorffer E, Mannes GA, Richter WO, Ochsenkuhn T, Seeholzer G, Kopcke W, Wiebecke B, Paumgartner G (1993). Decreased High-Density Lipoprotein Cholesterol and Increased Low-Density Cholesterol Levels in Patients with Colorectal Adenomas. Ann Intern Med.

[38] Poynter JN, Gruber SB, Higgins PD, Almog R, Bonner JD, Rennert HS, Low M, Greenson JK, Rennert G (2005). Statins and the risk of colorectal cancer. New Engl J Med.

[39] Murdocca M, Mango R, Pucci S, Biocca S, Testa B, Capuano R, Paolesse R, Sanchez M, Orlandi A, di Natale C, Novelli G, Sangiuolo F (2016). The lectin-like oxidized LDL receptor-1: A new potential molecular target in colorectal cancer. Oncotarget.

[40] Rizzacasa B, Morini E, Pucci S, Murdocca M, Novelli G, Amati F (2017). LOX-1 and Its Splice Variants: A New Challenge for Atherosclerosis and Cancer-Targeted Therapies. Int J Mol Sci.

[41] (2016). NCD Risk Factor Collaboration. Worldwide trends in diabetes since 1980: a pooled analysis of 751 population-based studies with 4.4 million participants. The Lancet.

[42] Parkin DM (2006). The global health burden of infection-associated cancers in the year 2002. Int J Cancer.

[43] Liang Q, Wendelhag I, Wikstrand J, Gustavsson T (2000). A multiscale dynamic programming procedure for boundary detection in ultrasonic artery images. IEEE Trans Med Imaging.

[44] Wendelhag I, Wiklund O, Wikstrand J (1993). Atherosclerotic changes in the femoral and carotid arteries in familial hypercholesterolemia: ultrasonographic assessment of intima-media thickness and plaque occurrence. Arterioscler Thromb.

[45] Winawer SJ, Zauber AG, Ho MN, O’Brien MJ, Gottlieb LS, Sternberg SS, Waye JD, Schapiro M, Bond JH, Panish JF, Ackroyd F, Shike M, Kurtz RC, the National Polyp Study Workgroup (1993). Prevention of colorectal cancer by colonoscopic polypectomy. The National Polyp Study Workgroup. N Engl J Med.

