# Supplementary material for: Hyperglycemia combined Helicobacter pylori infection increases risk of synchronous colorectal adenoma and carotid artery plaque
Source: Oncotarget. 2017 Oct 26;8(65):108655–64. doi: 10.18632/oncotarget.22094 (PMC5752471; doi:10.18632/oncotarget.22094)
Supplement: Supplementary file 1 [file oncotarget-08-108655-s001.pdf]

Hyperglycemia combined *Helicobacter pylori* infection increases risk of synchronous colorectal adenoma and carotid artery plaque

SUPPLEMENTARY MATERIALS

Supplementary Table 1A: Demoraphics of only colon adenoma compare to no adenoma and no carotid artery plaque

Variable	Only colon adenoma (*N* = 459)	No adenoma and No plaque (*N* = 1325)	*p*-value
Age, mean (SD), year	55.64 (9.46)	50.33 (9.75)	< .0001
Sex (male %)	324 (70.59)	796 (60.12)	< .0001
BMI, mean (SD), kg/m2	25.11 (3.52)	24.19 (3.41)	< .0001
HbA1c, mean (SD), %	5.85 (0.93)	5.71 (0.75)	0.0021
Systolic blood pressure, mean (SD) mm Hg	123.77 (15.50)	120.08 (37.58)	0.0035
Diastolic blood pressure, mean (SD) mm Hg	77.75 (9.51)	75.56 (19.62)	0.0017
Glucose AC, mean (SD), mg/dL	102.88 (24.51)	98.97(20.60)	0.0024
Total cholesterol, mean (SD), mg/dL	202.63 (39.49)	202.80 (36.14)	0.9358
Triglyceride, mean (SD), mg/dL	152.92 (106.48)	134.94 (80.96)	0.001
LDL, mean (SD), mg/dL	135.48 (36.64)	134.66 (33.96)	0.6749
Plasma hs-CRP > 0.3 mg/L, no. (%)	58 (20.94)	119 (16.08)	0.0689
WBC count, mean(SD), x 10^3/μL	6.49 (1.90)	6.17 (1.76)	0.0017
Smoking, no. (%)	129 (28.10)	281 (21.22)	0.0025
Anti-platelet agent used, no. (%)	23 (5.01)	51 (3.85)	0.2848
Anti-lipid agent used, no. (%)	65 (14.16)	118 (8.92)	0.0014
DM control agent used, no. (%)	33 (7.19)	97 (7.34)	0.9165
Hypertension control agent used, no. (%)	134 (29.19)	216 (16.35)	< .0001
Hb A_{1c} level ≥ 6.5%, no. (%)	68 (14.81)	97 (7.32)	< .0001
H. pylori-positive, no. (%)	204 (44.44)	414 (31.25)	< .0001

BMI: body mass index; $HbA_{1c,}$ glycated hemoglobin; LDL: Low-density lipoprotein; hs-CRP, high-sensitivity C-reactive protein DM: diabetes mellitus

Supplementary Table 1B: Demoraphics of only carotid artery plaque compare to no adenoma and no carotid artery plaque

Variable	Only carotid artery plaque (*N* = 353)	No adenoma and No plaque (*N* = 1325)	*p*-value
**Age, mean (SD), year**	56.93 (10.26)	50.33 (9.75)	< .0001
**Sex (male %)**	255 (72.24)	796 (60.12%)	< .0001
**BMI, mean (SD), kg/m2**	25.28 (10.25)	24.19 (3.41)	0.0504
**HbA1c, mean (SD), %**	5.97 (101)	5.71 (0.75)	< .0001
**Systolic blood pressure, mean (SD) mm Hg**	129.30 (68.89)	120.08 (37.58)	0.0161
**Diastolic blood pressure, mean (SD) mm Hg**	79.13 (34.53)	75.56 (19.62)	0.0636
**Glucose AC, mean (SD), mg/dL**	103.92 (25.83)	98.97(20.60)	0.0011
**Total cholesterol, mean (SD), mg/dL**	208.01 (35.90)	202.80 (36.14)	0.0164
**Triglyceride, mean (SD), mg/dL**	147.04 (78.55)	134.94 (80.96)	0.0124
**LDL, mean (SD), mg/dL**	140.85 (34.35)	134.66 (33.96)	0.0027
**Plasma hs-CRP > 0.3 mg/L, no. (%)**	46 (24.60)	119 (16.08)	0.0065
**WBC count, mean(SD), x 10^3/μL**	6.46 (1.89)	6.17 (1.76)	0.0072
**Smoking, no. (%)**	82 (23.23)	281/L(21.22%)	0.4161
**Anti-platelet agent used, no. (%)**	23 (6.52)	51 (3.85%)	0.0306
**Anti-lipid agent used, no. (%)**	53 (15.01)	118 (8.92%)	0.0008
**DM control agent used, no. (%)**	44 (12.57)	97 (7.34%)	0.0017
**Hypertension control agent used, no. (%)**	112 (32.00)	216 (16.35)	< .0001
**Hb A_{1c} level ≥ 6.5%, no. (%)**	57 (16.15)	97 (7.32%)	< .0001
***H. pylori*-positive, no. (%)**	162 (45.89)	414 (31.25%)	< .0001

BMI: body mass index; HbA_{1c}, glycated hemoglobin; LDL: Low-density lipoprotein; hs-CRP, high-sensitivity C-reactive protein DM: diabetes mellitus

Supplementary Table 2A: Univariate analysis and multivariate logistic regression for predictors of only colon adenoma ($N = 459$)

	Only colon adenoma	No adenoma and no plaque	Univariate analysis		Multivariate logistic regression	
			OR (95% CI)	*p*-value	Adjusted OR (95% CI)	*p*-value
**Age ≥ 60 years**	154	208	2.709 (2.123, 3.457)	< .0001	2.674 (1.889, 3.784)	<.0001
**Male, sex**	324	796	1.592 (1.266, 2.001)	< .0001	1.340 (0.961, 1.870)	0.0846
**BMI > 27**	128	234	1.802 (1.406, 2.309)	< .0001	1.611 (1.125, 2.307)	0.0093
**Systolic pressure ≥ 140 mm Hg**	83	148	1.756 (1.310, 2.353)	0.0002	1.362 (0.880, 2.107)	0.166
**Smoking**	129	281	1.451 (1.139, 1.849)	0.0026	1.496 (1.033, 2.107)	0.0329
**Anti-platelet agent used**	23	51	1.316 (0.795, 2.178)	0.286	0.555 (0.259, 1.189)	0.13
**Anti-lipid agent used**	65	118	1.685 (1.220, 2.329)	0.0016	1.377 (0.828, 2.292)	0.218
**HTN medicine**	134	216	2.109 (1.645, 2.704)	< .0001	1.043 (0.700, 1.554)	0.836
**DM control agent used**	33	97	0.978 (0.659, 1.474)	0.9168	0.179 (0.079, 0.406)	<.0001
**LDL > 130 mg/ dL**	234	694	0.950 (0.760, 1.164)	0.5705	1.015 (0.752, 1.369)	0.924
**WBC count x 10^3/μL**	458	1298	1.099 (1.038, 1.164)	0.0012	1.062 (0.976, 1.156)	0.1647
**hs CRP > 0.3 mg/L**	58	119	1.382 (0.974, 1.961)	0.0697	1.067 (0.723, 1.574)	0.7458
**Hb A_{1c} ≥ 6.5%**	68	97	2.202 (1.583, 3.063)	<.0001	3.439 (1.867, 6.333)	<.0001
***H. pylori*-positive**	204	414	1.761 (1.416, 2.189)	<.0001	1.966 (1.459, 2.651)	<.0001

BMI: body mass index; HbA_{1c}, glycated hemoglobin; LDL: Low-density lipoprotein; hs-CRP, high-sensitivity C-reactive protein DM: diabetes mellitus

Supplementary Table 2B: Univariate analysis and multivariate logistic regression for predictors of only carotid artery plaque ($N = 353$)

	Only Carotid artery plaque	No adenoma and no plaque	Univariate analysis		Multivariate logistic regression	
			OR (95% CI)	*p*-value	Adjusted OR (95% CI)	*p*-value
**Age ≥ 60 years**	146	208	3.784 (2.922, 4.900)	< .0001	2.962 (2.004, 4.378)	< .0001
**Male, sex**	255	796	1.726 (1.334, 2.233)	< .0001	2.071 (1.392, 3.081)	0.0003
**BMI > 27**	78	234	1.321 (0.990, 1.763)	0.0583	0.881 (0.567, 1.370)	0.5750
**Systolic pressure ≥ 140 mm Hg**	59	148	1.601 (1.154, 2.223)	0.0049	1.019 ((0.621, 1.672)	0.9406
**Smoking**	82	281	1.123 (0.849, 1.486)	0.4163	1.114 (0.720, 1.723)	0.6284
**Anti-platelet agent used**	23	51	1.738 (1.047, 2.886)	0.0325	1.066 (0.524, 2.170)	0.8600
**Anti-lipid agent used**	53	118	1.804 (1.274, 2.555)	0.0009	1.172 (0.669, 2.051)	0.5794
**HTN medicine**	112	216	2.407 (1.842, 3.147)	< .0001	1.349 (0.886, 2.051)	0.1628
**DM control agent used**	44	97	1.816 (1.245, 2.649)	0.0020	1.079 (0.553, 2.103)	0.8241
**LDL > 130 mg/ dL**	210	694	1.377 (1.082, 1.753)	0.0094	1.310 (0.923, 1.858)	0.1303
**WBC count x 10^3/μL**	347	1298	1.091 (1.024, 1.163)	0.0075	0.995 (0.900, 1.099)	0.9147
**hs CRP > 0.3 mg/L**	46	119	1.702 (1.157, 2.505)	0.0069	1.478 (0.954, 2.289)	0.0801
**Hb A_{1c} ≥ 6.5%**	57	97	2.438 (1.716, 3.462)	< .0001	1.932 (1.028, 3.633)	0.0409
***H. pylori*-positive**	162	414	1.866 (1.469, 2.371)	< .0001	1.419 (0.997, 2.022)	0.0523

BMI: body mass index; HbA_{1c}, glycated hemoglobin; LDL: Low-density lipoprotein; hs-CRP, high-sensitivity C-reactive protein DM: diabetes mellitus
